# Disrupted Lipid Raft Shuttling of FcεRI by n-3 Polyunsaturated Fatty Acid Is Associated With Ligation of G Protein-Coupled Receptor 120 (GPR120) in Human Mast Cell Line LAD2

**DOI:** 10.3389/fnut.2020.597809

**Published:** 2020-11-26

**Authors:** Xiaofeng Wang, Ramses Ilarraza, Brian P. Tancowny, Syed Benazir Alam, Marianna Kulka

**Affiliations:** ^1^Department of Agricultural, Food and Nutritional Science, University of Alberta, Edmonton, AB, Canada; ^2^Department of Medical Microbiology and Immunology, University of Alberta, Edmonton, AB, Canada; ^3^Department of Biochemistry, Prion Research Institute, University of Alberta, Edmonton, AB, Canada; ^4^National Research Council Canada, Nanotechnology Research Centre, Edmonton, AB, Canada

**Keywords:** n-3 PUFA, mast cells, FcεRI, GPR120, lipid rafts, GPR40 and inflammation

## Abstract

n-3 polyunsaturated fatty acids (PUFA) influences a variety of disease conditions, such as hypertension, heart disease, diabetes, cancer and allergic diseases, by modulating membrane constitution, inhibiting production of proinflammatory eicosanoids and cytokines, and binding to cell surface and nuclear receptors. We have previously shown that n-3 PUFA inhibit mast cell functions by disrupting high affinity IgE receptor (FcεRI) lipid raft partitioning and subsequent suppression of FcεRI signaling in mouse bone marrow-derived mast cells. However, it is still largely unknown how n-3 PUFA modulate human mast cell function, which could be attributed to multiple mechanisms. Using a human mast cell line (LAD2), we have shown similar modulating effects of n-3 PUFA on FcεRI lipid raft shuttling, FcεRI signaling, and mediator release after cell activation through FcεRI. We have further shown that these effects are at least partially associated with ligation of G protein-coupled receptor 120 expressed on LAD2 cells. This observation has advanced our mechanistic knowledge of n-3 PUFA's effect on mast cells and demonstrated the interplay between n-3 PUFA, lipid rafts, FcεRI, and G protein-coupled receptor 120. Future research in this direction may present new targets for nutritional intervention and therapeutic agents.

## Introduction

As precursors of eicosanoids and important constituents of lipid membranes, n-3 polyunsaturated fatty acids (PUFA) especially eicosapentaenoic acid (EPA, 20:5, n-3) and docosahexaenoic acid (DHA, 22:6, n-3) have significant impacts on inflammatory diseases. n-3 PUFA may play important roles in various diseases such as rheumatoid arthritis, inflammatory bowel disease, asthma, cancer, diabetes, cardiovascular disease, neurodegenerative disease, hyperlipidemia, and hypertension (1–7). EPA and DHA are concentrated in neural tissues including brain and retina as well as in human milk, plasma, mucosa, and sperm (2, 8–11).

One of the major beneficial properties of n-3 PUFA is their action as anti-inflammatory molecules (12). n-3 PUFA reduce the production of pro-inflammatory mediators by competitively inhibiting the synthesis of eicosanoids from arachidonic acid (AA, 20:4, n-6) and suppressing the gene transcription of pro-inflammatory cytokines through binding to cell surface G protein-coupled receptor (GPR) 40 and GPR120, also called free fatty acid receptors 1 and 4 (FFA1 and FFA4), as well as nuclear peroxisome proliferator-activated receptors (PPAR) (2). Previous work from our lab suggests that n-3 PUFA modulate the response of prostate cancer cells to interferon-γ and inhibit the production of interleukin-18 binding protein, which neutralizes interleukin-18 (13). Both EPA and DHA can be metabolized to produce anti-inflammatory molecules also called resolvins and protectins, capable of dampening inflammation (12, 14). Since n-3 PUFA have been associated with alleviated mucosal inflammation in the context of inflammatory bowel disease and the airway mucosa is also enriched with n-3 PUFA (10, 11), these molecules could potentially play an anti-inflammatory function in airway inflammatory diseases.

Studies in both asthmatic patients and *in vivo* animal models of allergic asthma have demonstrated the association between n-3 PUFA intake and reduced bronchial inflammation (15–17). There is an association between plasma n-3 PUFA levels and a reduced risk of developing allergic diseases (18). In addition, erythrocytes from asthmatic patients have a significantly higher ratio of n-6/n-3 PUFA in their membranes compared to healthy subjects, which was associated with higher plasma concentrations of proinflammatory n-6 PUFA-derived eicosanoids (19).

Mast cells (MCs) are one of the key effector cells that mediate allergic inflammatory responses (20), and are canonically activated by IgE/allergen crosslinking of the high affinity IgE receptors (FcεRI) during allergic responses (21). There is experimental evidence of a modulatory effect of n-3 PUFA on MC functions. Docosahexaenoyl ethanolamide, a DHA metabolite, supresses degranulation of rat basophilic leukemia RBL-2H3 cells and bone marrow-derived mast cells (22). Our previous study using mouse bone marrow-derived mast cells suggests that n-3 PUFA inhibit mast cell activation by suppressing FcεRI-mediated signal transduction and disrupting FcεRI shuttling to lipid rafts, which are specialized membrane microdomains with concentrated localization of transmembrane proteins, sphingolipids, and cholesterol (23). Furthermore, other groups have also shown that n-3 PUFA inhibit canine and rodent mast cell lines activated by G protein agonists and calcium ionophore (24–27), indicating that n-3 PUFA may modulate both FcεRI- and non-FcεRI-mediated pathways in mast cell activation. However, the mechanism by which n-3 PUFA exert their effects on FcεRI-mediated activation and the subsequent effects on degranulation and leukotriene production are still poorly understood.

In this study, we hypothesized that n-PUFA modulates FcεRI-mediated activation of human mast cells by disrupting the localization of FcεRI within lipid rafts, essential for effective FcεRI-mediated phosphorylation of downstream signaling molecules such as the spleen tyrosine kinase (Syk) and linker for activated T cells (LAT). Disruption of this membrane-proximal event thereby leads to dampened degranulation and cytokine release. We examined the effect of n-3 PUFA on FcεRI localization and signaling, as well as mediator release using the LAD2 (Laboratory of Allergic Diseases 2) human mast cell line. Since n-PUFA are known to activate GPR40 and GPR120, we examined the role of these receptors in mast cell degranulation, showing for the first time that these receptors are expressed by human mast cells and have a functional role in mast cell mediator release.

## Materials and Methods

### Reagents and Cell Culture

All reagents used in this study were obtained from Sigma-Aldrich (Oakville, Canada) unless stated otherwise. LAD2 human mast cells were cultured in serum-free StemPro-34 SFM medium (Thermo Fisher Scientific, Waltham, MA, USA) containing 2 mM L-glutamine, 100 U/ml penicillin, and 50 μg/ml streptomycin. Stem cell factor (SCF, Peprotech Inc, Rocky Hill, USA), was added to the media at 100 ng/ml, which is required for optimal mast cell differentiation and proliferation (28). Cells were seeded at 1 × 10^5^ cells/mL and maintained at 37°C and 5% CO_2_. Cells were fed by hemi-depletion of culture media every week, and periodically tested for the expression of FcεRI and Kit by flow cytometry. For experiments involving mast cell activation, cells were incubated with biotinylated IgE (BioLegend, San Diego, USA; 500 ng/ml) for at least 18 h, followed by activation with streptavidin (SA) for 30 min, which results in strong and consistent degranulation (29).

### Cell Viability Assay

LAD2 cells were cultured with increasing concentrations (0.1, 1, 10, and 100 μM) of EPA sodium salt (EPA-Na) and DHA sodium salt (DHA-Na) for 24 and 48 h. Cell viability was measured using the 2,3-bis-(2-methoxy-4-nitro-5-sulfophenyl)-2H-tetrazolium-5-carboxanilide (XTT) cell proliferation kit (Roche Molecular Biochemicals, Indianapolis, USA) according to the manufacturer's instructions.

### Degranulation Assay

LAD2 cells were treated with 10 μM GW9508 (a GPR40/120 agonist) and 10 μM TUG891 (a GPR120 agonist) for 24 h or with 10, 50, and 100 μM EPA-Na and DHA-Na with or without 30 min pre-treatment with 10 μM AH7614, a GPR120 antagonist. Cells were then sensitized with 500 ng/mL IgE-biotin (BioLegend, San Diego, USA) for 24 h in culture media and stimulated with 500 ng/ml SA in HEPES buffer for 30 min. Mast cell degranulation was assessed by measuring β-hexosaminidase (β-hex) release with a method described previously (30).

### qPCR Analysis

Total RNA was isolated from LAD2 cells with TRI reagent, followed by cDNA synthesis using a first strand cDNA synthesis kit (Thermo Fisher Scientific). SYBR green quantitative PCR Mastermix (Thermo Fisher Scientific) was used for quantitative PCR amplification using the Rotor gene multiplex HRM thermal cycler (Qiagen, Germantown, USA) with GAPDH as the reference gene. The primer sets for human GPR40 (reverse primer: 5′ GGA TTA AGC ACC ACA CTC CA 3′; forward primer: 5′ TCT CCT TCG GCC TCT ATG T 3′), GPR120 (reverse primer: 5′ GAT GAG GAG GAT GGT GAT GAT G 3′; forward primer: 5′ CCG ACC AGG AAA TTT CGA TTT G 3′) and GAPDH (forward primer: 5′ ACC ACA GTC CAT GCC ATC AC 3′; Reverse primer: 5′ TCC ACC ACC CTG TTG CTG TA 3′) were designed using Primer-BLAST. The data were normalized and analyzed using the 2^−ΔΔCt^ method (31).

### Enzyme-linked Immunosorbent Assay (ELISA) Measurement of Cysteinyl Leukotrienes (CysLT)

LAD2 cells were treated with 100 μM EPA-Na and DHA-Na for 24 h, followed by 24 h sensitization with 500 ng/mL IgE-biotin and stimulation with 500 ng/ml SA for 6 h. Supernatants were collected by centrifugation and analyzed for CysLT release using commercial ELISA kits (Enzo Life Sciences, Farmingdale, USA), according to the manufacturer's protocols.

### Flow Cytometry

Briefly, cells were washed with phosphate-buffered saline (PBS) containing 0.1% BSA and re-suspended in PBS containing 1.5% BSA to obtain 2 × 10^6^ cells/mL, and stained with 1 μg/mL mouse anti-human FcεRIα PE (eBioscience, San Diego, USA) for 1 h. Mouse IgG PE (eBioscience) was used as an isotype control. After washing twice with 0.1% BSA in PBS, cells were resuspended in 200 μl 0.1% BSA in PBS and transferred to a round bottom 96-well-plate. Cell samples were analyzed on a FACS Array flow cytometer (BD Biosciences, Mississauga, Canada). Data were generated using WinMDI 2.9 software (Joe Trotter, The Scripps Research Institute, La Jolla, USA). Results are presented as mean fluorescence intensity.

### Lipid Raft Isolation

Lipid rafts were isolated by sucrose gradient centrifugation as previously described (23) using a protocol modified from *Brown* (32). Briefly, cell lysates of 2 × 10^8^ cells were mixed with ice-cold TNE buffer (1% Triton X-100) containing 80% (w/v) sucrose before transferring to an ultracentrifuge tube (Beckman Coulter, Mississauga, Canada). Cell lysate/80% sucrose mixtures were overlaid with ice-cold TNE buffer containing 35% (w/v) sucrose, followed by ice-cold 5% (w/v) sucrose in TNE buffer. After centrifugation for 3 h at 100,000 × g at 4°C, a visible band of lipid raft fraction was harvested at the 5%/35% sucrose interface.

### Western Blot

Electrophoresis was performed on whole cell protein and lipid raft fractions using NuPage Novex 4–12% Bis-Tris Gels (Thermo Fisher Scientific) and nitrocellulose membranes (Thermo Fisher Scientific) with a method described previously (33). Membranes were then blocked with 5% non-fat milk/TBS-T at 4°C overnight, followed with primary antibody incubation overnight. The antibodies used were rabbit polyclonal anti-human GPR120 antibody (Novus Biologicals, Centennial, CO, USA), rabbit polyclonal anti-human Lyn, pho-Lyn, spleen tyrosine kinase (Syk), pho-Syk, linker of activated T cells (LAT), and pho-LAT antibodies (Cell Signaling Technology, Danvers, USA), rabbit polyclonal anti-human FcεRIα (Santa Cruz Biotechnology, Santa Cruz, USA) and rabbit monoclonal anti-actin antibodies. Images were obtained in a ChemiDoc XRS system (BD Biosciences) after incubation with secondary antibody horseradish peroxidase-conjugated goat anti-rabbit IgG (Santa Cruz Biotechnology) and chemiluminescent peroxidase substrate.

### Statistical Analysis

The differences between groups were evaluated by one-way analysis of variance (ANOVA) followed by the Bonferroni *post-hoc* test. Statistical significance was set at *p* < 0.05. Statistical analyses were performed using GraphPad prism (GraphPad Software, San Diego, USA).

## Results

### n-3 PUFA Reduce FcεRI Subcellular Localization to Lipid Rafts, but Do Not Affect Surface or Cellular Expression of FcεRI

**Figure 1:**
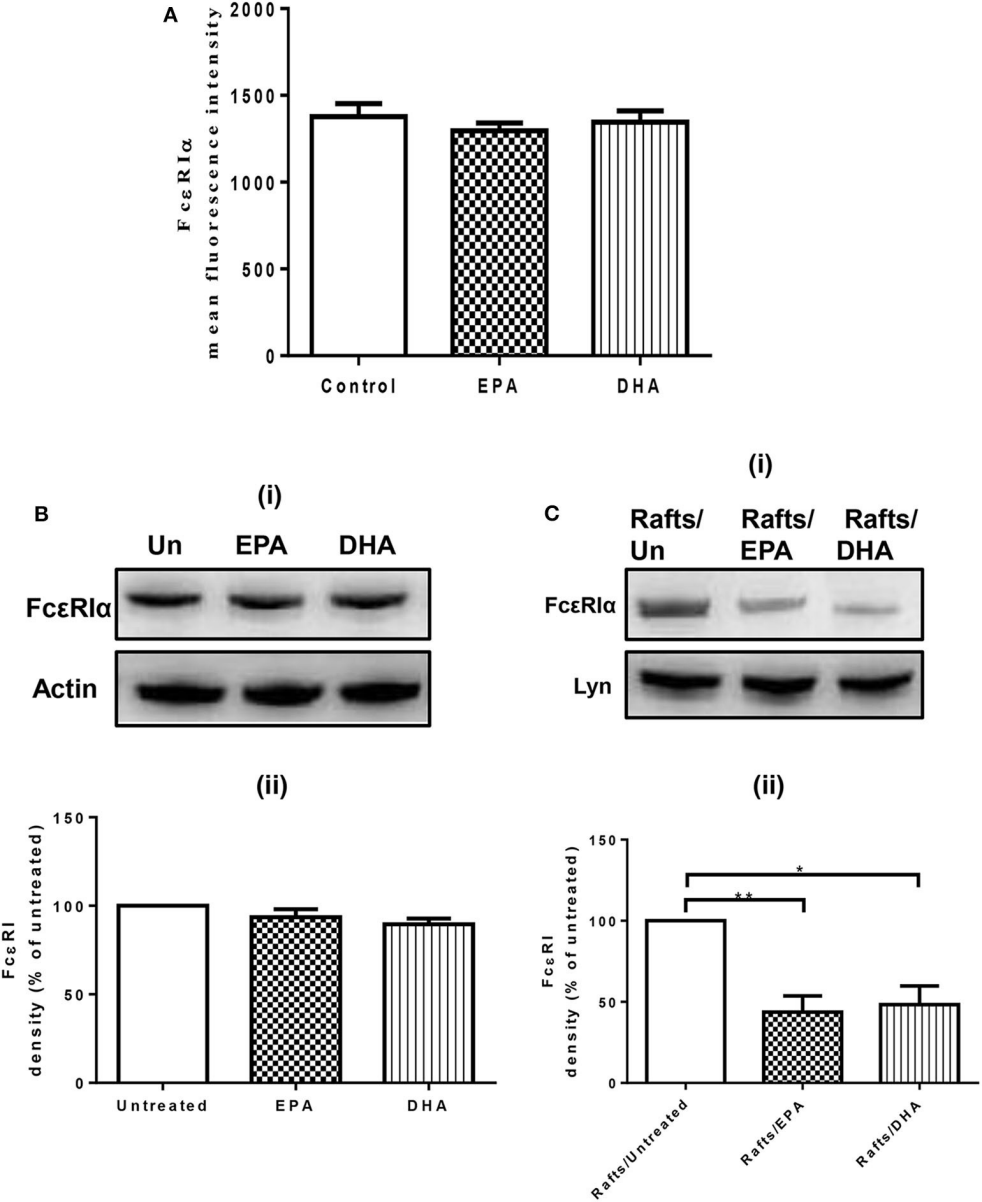
n-3 PUFA reduce FcεRI subcellular localization to lipid rafts, but do not affect surface or cellular expression of FcεRI. **(A)** Flow cytometry analysis of FcεRI expression on the cell surface of LAD2 cells after 24 h treatment with 100 μM EPA-Na and DHA-Na. **(B,C)** Western blot (i) and densitometric (ii) analyses of FcεRI in whole cell lysates **(B)** and lipid raft fractions **(C)** using actin and Lyn as loading controls, respectively. *p < 0.05, **p < 0.01, Error bars represent SEM (*n* = 3–4).

To evaluate the possibility that n-3 PUFA directly modify FcεRI expression, we examined cell surface expression of FcεRI on EPA and DHA-treated LAD2 cells by flow cytometry. EPA-Na (referred to as EPA henceforth) and DHA-Na (referred to as DHA henceforth) did not alter FcεRI expression (Figure 1A). To determine whether n-3 PUFA modified cellular expression of FcεRI, we examined FcεRI expression in whole cell lysates of LAD2 cells treated with EPA and DHA. Neither EPA nor DHA altered the cellular expression of FcεRI (Figure 1B). We further determined the relative distribution of receptor on lipid rafts, cholesterol-rich lipid microdomains that play an important role in MC signaling events on the plasma membrane. Supplementation of LAD2 cells with 100 μM EPA and DHA for 24 h significantly reduced FcεRI partitioning in lipid rafts (by 56 and 52%, respectively) when compared to untreated cells (Figure 1C). These results show that n-PUFA do not affect expression of FcεRI, but alter its distribution in lipid rafts.

### n-3 PUFA Suppress Phosphorylation of Lyn, Syk, and LAT Activated by IgE-crosslinking

**Figure 2:**
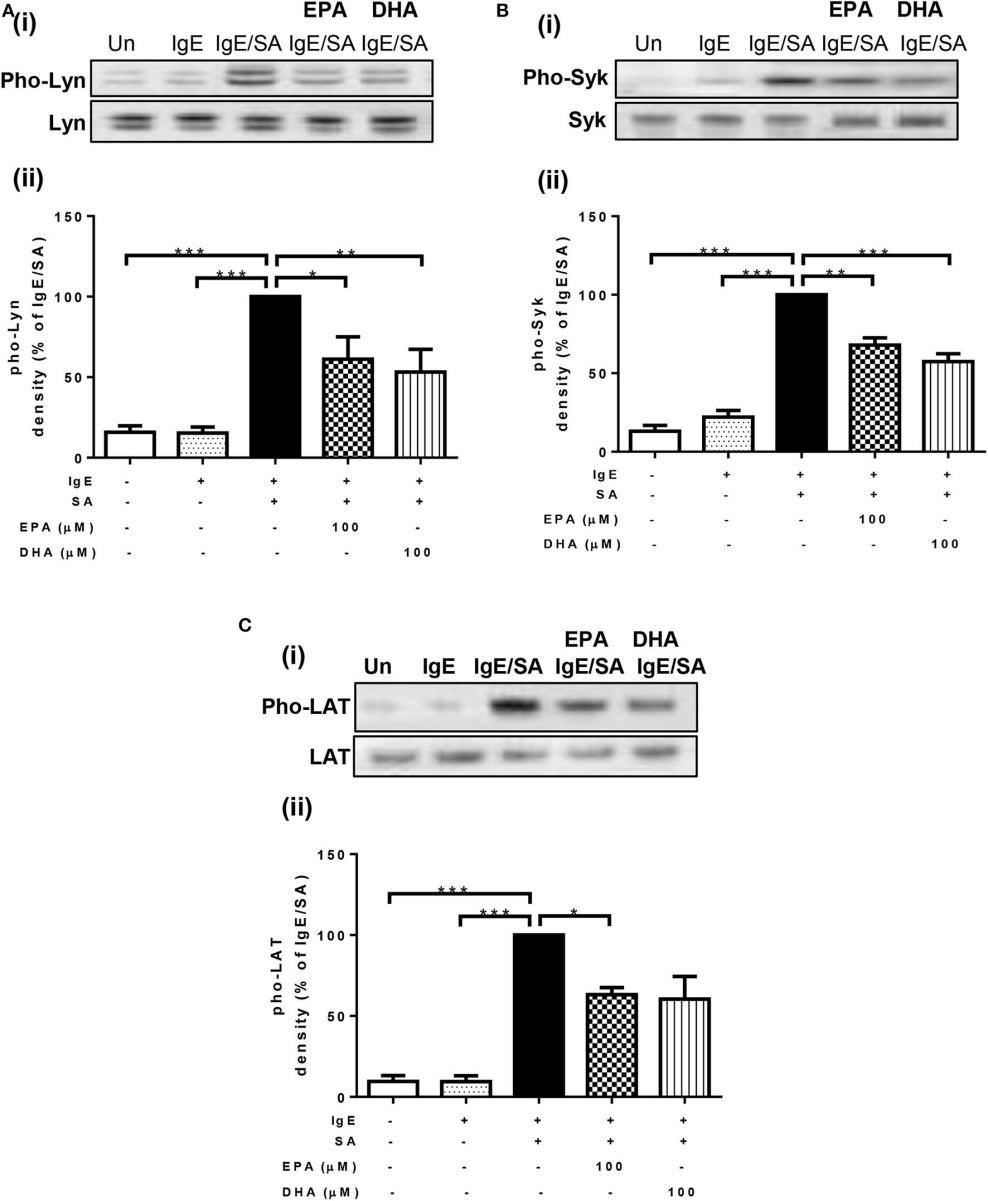
n-3 PUFA suppress phosphorylation of Lyn, Syk, and LAT activated by IgE-crosslinking. Western blot (i) and densitometric (ii) analyses of phosphorylated (Phos, Western blot in upper panel) and unphosphorylated (Western blot in lower panel) isoforms of Lyn **(A)**, Syk **(B)**, and LAT **(C)** proteins in LAD2 cells pre-treated with 100 μM EPA-Na (EPA) or DHA-Na (DHA) for 24 h, followed with sensitization with 500 ng/mL IgE-biotin for 24 h, and stimulation with 500 ng/mL SA for 30 min. Densitometric analysis of phosphorylated protein expression levels was standardized relative to that of IgE/SA samples and is represented at a percentage. *p < 0.05, **p < 0.01, ***p < 0.001; Error bars represent SEM (*n* = 3–4).

To identify the mechanism through which n-3 PUFA reduce FcεRI-mediated signaling, we first measured their effects on the phosphorylation state of three important signal transduction proteins (Lyn, Syk, and LAT) that are normally phosphorylated shortly after FcεRI-crosslinking. For this purpose, LAD2 cells were cultured with 100 μM EPA and DHA, and then stimulated with IgE-biotin/SA. As expected, IgE-biotin and SA activation resulted in phosphorylation of Lyn, Syk, and LAT compared to cells treated only with IgE-biotin (6.6-, 4.5-, and 11.6-fold increase, respectively; Figures 2A–C). Pre-treatment of LAD2 cells with EPA and DHA significantly reduced the phosphorylation of Lyn (Figure 2A) and Syk (Figure 2B), and LAT (Figure 2C) when the cells were stimulated with SA for 30 min, with the exception of LAT phosphorylation, for which inhibition was not significant when cells were treated with 100 μM DHA. No significant difference in the phosphorylation levels of Lyn, Syk, and LAT was observed when lower concentrations of EPA and DHA were used (10 μM, data not shown), suggesting a threshold effect.

### n-3 PUFA Inhibit FcεRI-mediated Mediator Release by LAD2 Cells

**Figure 3:**
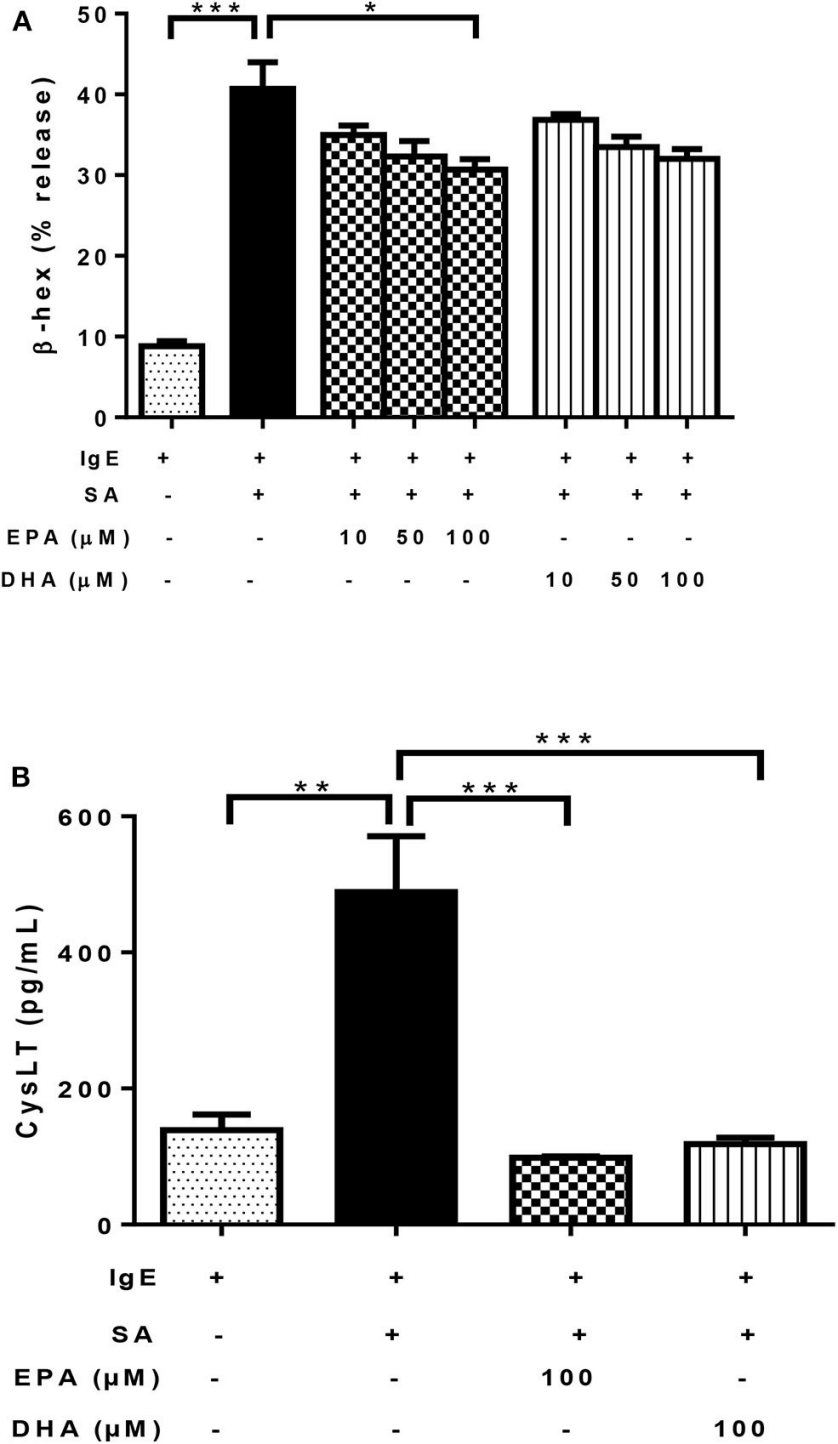
n-3 PUFA inhibit FcεRI-mediated mediator release by LAD2 cells. Percentage of β-hex **(A)** release from LAD2 cells pre-treated with increasing concentrations (10, 50, and 100 μM) of EPA-Na (EPA) and DHA-Na (DHA) for 24 h, followed with sensitization with 500 ng/mL IgE-biotin for 24 h and stimulation with 500 ng/mL SA for 30 min. **(B)** CysLT release in pg/ml (determined by ELISA) from LAD2 cells pre-treated with 100 μM EPA-Na (EPA) and DHA-Na (DHA) for 24 h, followed with sensitization with 500 ng/mL IgE-biotin for 24 h and stimulation with 500 ng/mL SA for 6 h. *p < 0.05, **p < 0.01, ***p < 0.001; Error bars represent SEM (*n* = 3–6).

As expected, the addition of SA to IgE-biotin-sensitized LAD2 cells significantly induced β-hexosaminidase (β-hex) release, compared to IgE sensitization alone (4.6-fold; Figure 3A). Treatment with both EPA and DHA decreased β-hex release in the range tested (10–100 μM, Figure 3A), but only 100 μM EPA reached statistical significance (25% reduction; Figure 3A) statistical significance for 100 μM EPA (25% reduction; Figure 3A). DHA showed a similar trend, but there was no statistical significance compared to IgE-biotin/SA-stimulated cells (*p* = 0.09 for 100 μM; Figure 3A).

As expected, activation of LAD2 cells using IgE-biotin and SA significantly increased CysLT production in LAD2 cells compared to IgE-biotin-sensitized cells (3.5-fold; Figure 3B). However, treatment with 100 μM EPA and DHA for 24 h significantly suppressed IgE-biotin/SA-induced CysLT secretion compared to stimulated cells (by 80 and 76%, respectively; Figure 3B). There was no statistically significant difference in CysLT release between EPA and DHA (Figure 3B). Since the observed differences with EPA and DHA could be due to a decrease in cell viability, we tested LAD2 cell viability with EPA and DHA at 0.1–100 μM for 24 and 48 h, and observed no effect on LAD2 cell viability (Supplementary Figure 1). We also measured TNF release by LAD2 cells after activation by ELISA (data not shown), but the observed levels of this cytokine were below the level of detection of the assay.

### n-3 PUFA's Inhibitory Effect on Mast Cell Degranulation Is Mediated via GPR120

**Figure 4:**
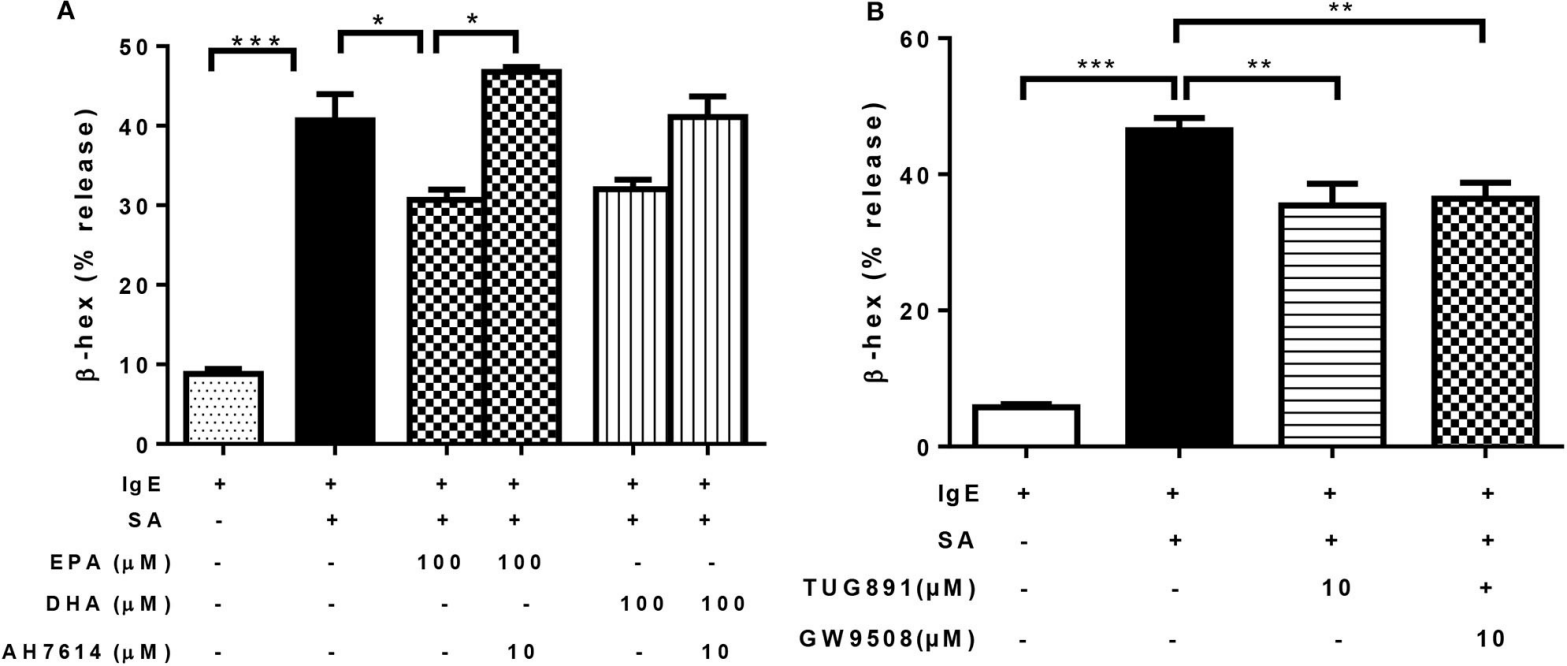
n-3 PUFA's inhibitory effect on mast cell degranulation is mediated via GPR120. **(A,B)** Percentage of β-hex release by LAD2 cells. Cells were treated with indicated concentrations of TUG891 (a GPR120 agonist), GW9508 (a GPR40/120 agonist), EPA-Na, and DHA-Na for 24 h with or without 30 min pre-treatment of GPR120 antagonist AH7614, which was followed by sensitization with 500 ng/mL IgE-biotin for 24 h and stimulation with 500 ng/mL SA for 30 min. *p < 0.05, **p < 0.01, ***p < 0.001; Error bars represent SEM (*n* = 3–6).

Previous studies have shown that EPA and DHA are ligands of GPR40 and GPR120, which have a regulatory role in insulin secretion and pro-inflammatory cytokine production (34, 35). It was possible that the inhibitory effect of n-3 PUFA on FcεRI-mediated degranulation was mediated via activation of GPR40 and/or GPR120. To test this, we first treated LAD2 cells with EPA in combination with GPR120 antagonist AH7614, prior to IgE-biotin/SA stimulation. AH7614 completely reverted the inhibitory effect of EPA on β-hex release, and a similar trend was observed for DHA, (Figure 4A). These results suggest that GPR120 could be involved in n-3 PUFA-mediated FcεRI-dependent LAD2 human MC activation.

We tested other GPR40 and GPR120 agonists to determine if they could exert similar effects on LAD2 cell degranulation. Both TUG891 (a GPR120 agonist) and GW9508 (an agonist for both GPR40 and 120) at 10 μM significantly suppressed β-hex release in stimulated LAD2 cells (by 24 and 22%; Figure 4B), which appear to confirm a role for GPR120.

### LAD2 Human Mast Cells Express GPR40 and GPR120

Next, we hypothesized that the free fatty acid inhibitory receptors GPR40 and GPR120 could be involved in the effects of n-3 PUFA that we observed. PUFA can activate GPR40 (FFAR1) and GPR120 (FFAR4) (36–38), leading to increases in intracellular cyclic adenosine monophosphate (cAMP), activation of protein kinase A, and inhibition of MC degranulation (39–41). Since expression of GPR40 and GPR120 by human MCs has not been described to date, we analyzed the expression of these receptors using both qPCR and western blot analysis.

**Figure 5:**
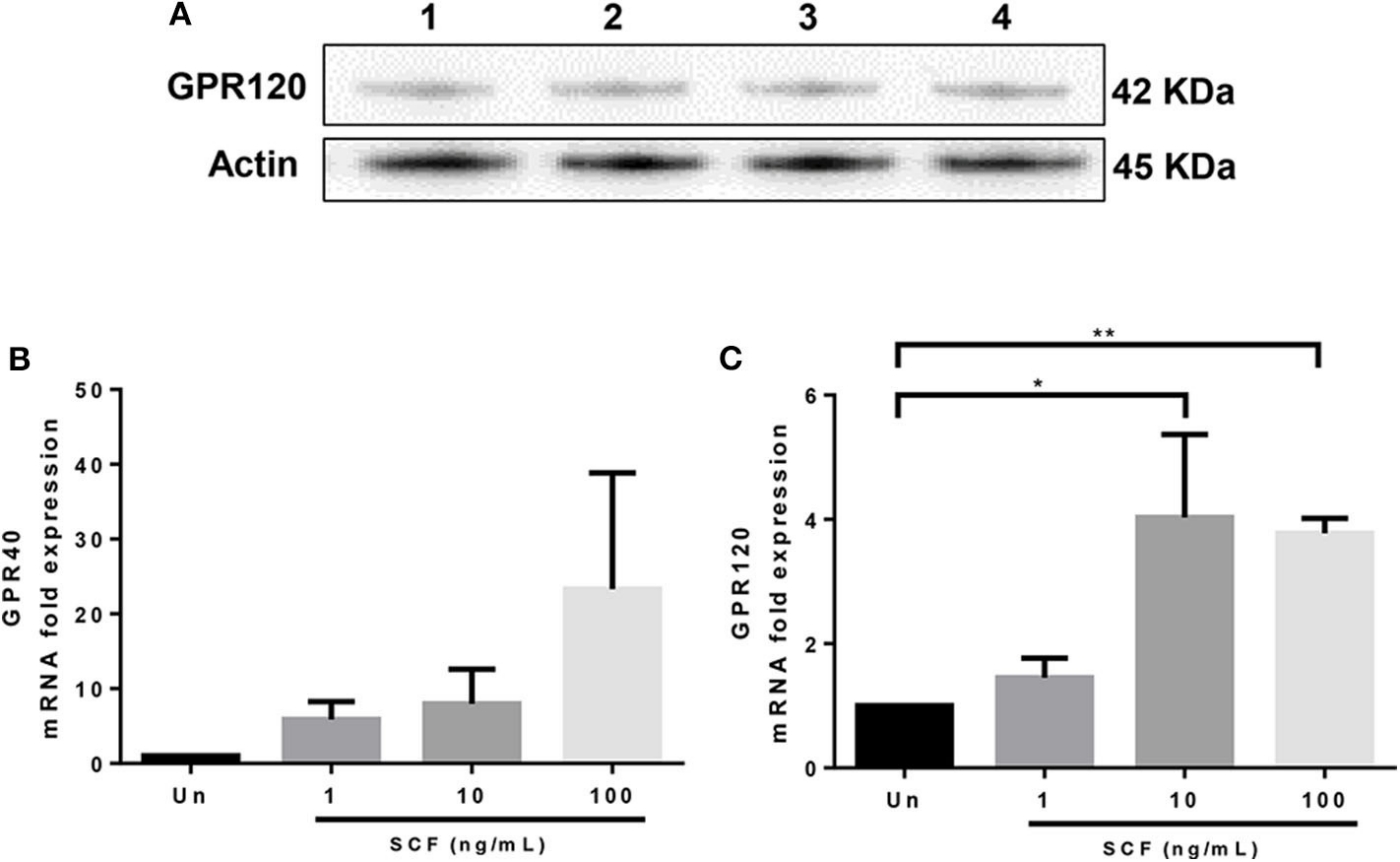
LAD2 human mast cells express GPR40 and GPR120. **(A)** Western blot analyses of GPR120 expression in LAD2 mast cells. Lanes 1–4 represent 4 replicates. **(B,C)** qPCR measurement of *GPR40* and *GPR120* mRNA expression normalized to *GAPDH* in LAD2 cells cultured in media with and without SCF (1–100 μM). *p < 0.05, **p < 0.01, Error bars represent SEM (*n* = 3–7).

Although GPR40 protein expression was not detectable by western blot analysis using the available antibody (data not shown), GPR120 protein expression was readily observed in LAD2 cells (Figure 5A). qPCR analysis confirmed that LAD2 cells express *GPR40* and *GPR120* mRNA and that SCF increased expression of both *GPR40* and *GPR120* mRNA in LAD2 human MCs (Figures 5B,C), in a dose-dependent manner (4- and 3.8-fold for 10 and 100 ng/ml, respectively; Figure 5C).

## Discussion

**Figure 6:**
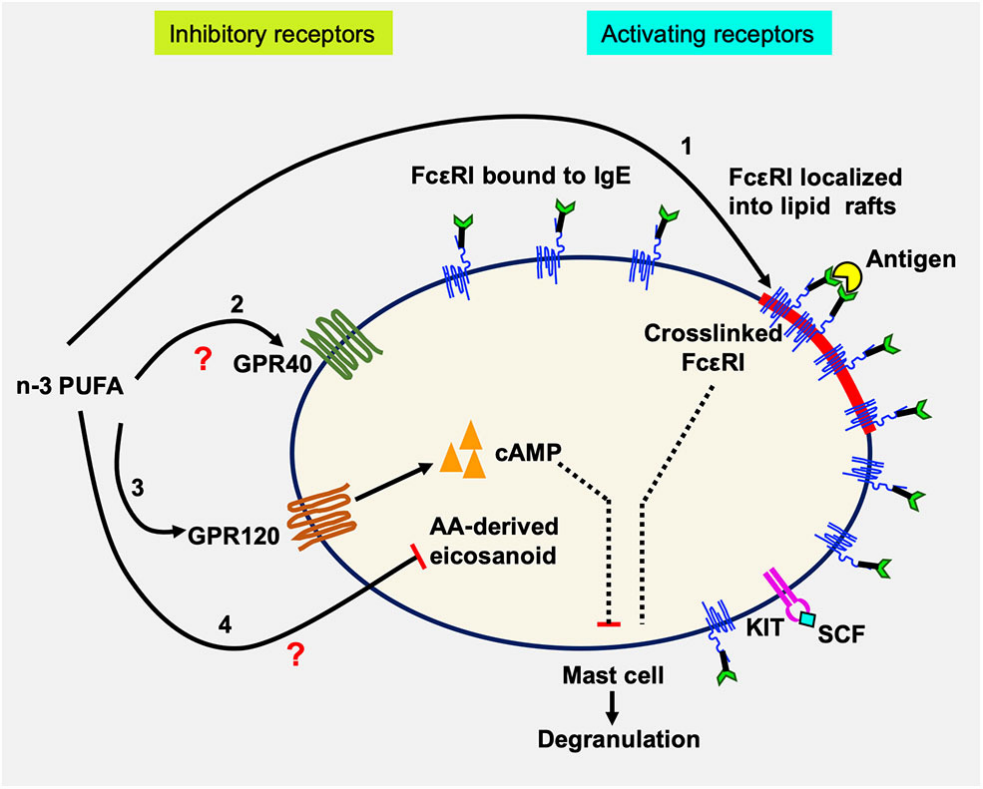
Schematic diagram showing possible actions of n-3 PUFA on LAD2 human MC. Human mast cells are complex immune cells which express both activating (FcεRI and KIT) and inhibitory receptors (GPR40 and GPR120), that must respond to stimuli in a balanced way. n-3 PUFAs can modulate FcεRI-mediated mast cell mediator release by altering lipid raft function especially by altering its localization into lipid rafts (1). In resting cells, the majority of FcεRI is bound to IgE but is excluded from lipid rafts. Upon IgE crosslinking by antigen, FcεRI is sequestered within lipid rafts where it is phosphorylated and initiates degranulation and mediator release. n-3 PUFAs can bind to and activate GPR40 (2) and GPR120 (3), which in turn could increase the concentration of cAMP which would suppress mast cell degranulation. It is also possible that n-3 PUFA inhibits the production of AA-derived eicosanoids by competitive inhibition (4). LAD2, like tissue-resident mast cells, are maintained in SCF which promotes their proliferation. SCF binding to KIT causes changes in *GPR40* and *GPR120* mRNA expression, possibly maintaining an important balance between the inhibitory and activating receptors.

In this study, we examined the effects of n-3 PUFA on IgE-mediated MC activation in LAD2 human MCs. The results showed that EPA and DHA reduced FcεRI localization into lipid rafts, but did not affect its surface or cellular expression. This was accompanied by decreased Lyn, Syk, and LAT phosphorylation, indicating inhibited FcεRI lipid raft localization and downregulated FcεRI signaling molecule phosphorylation. We further showed that EPA and DHA supplementation inhibited LAD2 human MC degranulation (β-hex release) and reduced production of lipid-derived mediators (CysLT) activated by FcεRI crosslinking (42). Interestingly, we found that inhibition of degranulation was reversed by an antagonist to n-3 PUFA receptor GPR120 (AH7614). We confirmed the expression of GPR120 in LAD2 cells, at mRNA and protein levels. Additionally, GPR120 ligation with agonists TUG891 and GW9508 resembled the inhibitory effects of n-3 PUFA on FcεRI-activated LAD2 degranulation, suggesting that GPR120 is a functional receptor on human MCs, and that GPR120 may play a role in the observed n-3 PUFA inhibition of human MC activation. Whether the effects of GPR120 occur directly on FcεRI localization to lipid rafts, signaling, or both, is still unknown. A schematic diagram summarizing possible mechanisms of n-3 PUFA's modulating effect on human MC activation is shown in Figure 6.

Lipid rafts are cholesterol-rich membrane microdomains found in plasma membranes and some intracellular membranes such as the Golgi complex (43, 44). The content of saturated fatty acids is higher in lipid rafts as compared to other regions (43), which results in a liquid-ordered state allowing them to facilitate rapid signaling events. Lipid rafts typically contain increased densities of molecules required for signaling and a more stable membrane local environment, making them an ideal platform for FcεRI-mediated signal transduction (43, 45), especially considering that the signal transduction initiated by FcεRI is very rapid. There is evidence that FcεRI localizes preferentially in lipid rafts in MCs (46). In addition, rafts are not fixed structures and can move and fuse with others, helping to efficiently aggregate FcεRI (47). Thus, the localization of FcεRI in lipid rafts is vital for signaling initiation, which is subject to change as a result of alterations in their fatty acid profile. Treatment with EPA and DHA has been shown to increase EPA and DHA levels in both raft and non-raft regions in MCs (48). Consistent with our previous study on mouse bone-marrow derived mast cells (23), we have shown that in LAD2 cells EPA and DHA supplementation decreased FcεRI partitioning to lipid rafts, which might be responsible for the decrease in FcεRI signal transduction as observed by diminished phosphorylation of Lyn, Syk, and LAT.

Lyn, Syk, and LAT are three essential signaling proteins in FcεRI-mediated activation, whose phosphorylation are largely dependent on FcεRI crosslinking (49, 50). The cross-linking of IgE-bound FcεRI with multivalent antigen results in the phosphorylation of immunoreceptor tyrosine-based activation motifs (ITAMs) by Lyn kinase, which is thought to be autophosphorylated. Phosphorylated ITAMs recruit and activate Syk (51), which in turn activate protein kinase C, leading to degranulation and the activation of the transcription factor NF-κB. Syk also phosphorylates LAT, which is an adaptor protein that mediates the activation of phospholipase C-γ and growth factor receptor-bound protein (49), which results in the generation of second messenger molecules, an increase in intracellular Ca^2+^ levels, and the activation of the mitogen-activated protein kinase pathway (52). The activation of mitogen-activated protein kinase pathway is responsible for the release of lipid derived mediators by activating cytosolic phospholipase A2. Activation of NF-κB, together with MAPK pathways, result in the transcription of cytokines and chemokines. Thus, Lyn, Syk, and LAT play critical roles in the initiation and subsequent amplification of FcεRI-mediated signaling.

FcεRI-mediated signaling in MCs leads to the gradual release of three groups of mediators which include preformed mediators (degranulation), lipid-derived mediators, and cytokines/chemokines, which are associated with inflammation (53). The preformed mediators such as β-hexosaminidase, histamine, proteases, and serotonin are stored in granules and released within seconds following cell activation (54). Our results showed that EPA inhibited the release of β-hexosaminidase, indicating reduced MC degranulation. These results are consistent with previous studies in rodent MCs (22, 23).

Eicosanoids which include prostaglandins, thromboxanes, and leukotrienes are released within minutes from MC after activation through FcεRI and play an essential role in the pro-inflammatory response. Eicosanoids are produced in reactions catalyzed by cyclooxygenase and lipoxygenase from n-3 (EPA and DHA) and n-6 (AA and dihomo-γ-linolenic acid) PUFA, which are freed from the plasma and nuclear membrane by cytoplasmic phospholipase A_2_ after cell activation (55). It is important to note that n-3 and n-6 PUFA are precursors to different classes of eicosanoids, and that eicosanoids synthesized from n-3 PUFA have less pro-inflammatory potentials than those (such as CysLT) from n-6 PUFA (50). In this study, we showed that treatment with EPA and DHA reduced CysLT release, which is consistent with the observation that EPA and DHA are not precursors for CysLT, rather they compete for the same enzymes required for the biosynthesis of eicosanoid from AA. Therefore, supplementation of EPA and DHA results in decreased CysLT production from AA.

The down-stream activation of transcription factors in FcεRI-mediated signaling causes the transcription and production of cytokines/chemokines, which are detected hours after cell activation. These mediators also play critical roles in the development of inflammation in allergic responses (56). In this study, we did not find increased cytokine production, at least in the form of TNF, after IgE-biotin/SA stimulation by LAD2 cells, possibly due to suboptimal crosslinking of FcεRI. IgE-biotin/SA only crosslinks four receptors whereas IgE/antigen would likely crosslink hundreds of FcεRI receptors.

GPR40 and GPR120 are functional receptors for medium and long chain free fatty acids with a preference to PUFA, including n-3 PUFA (36–38). GPR40 has been shown to alleviate inflammation, promote insulin secretion, enhance pancreatic β-cell survival, and regulate energy homeostasis (57–59). GPR120 enhances insulin sensitivity, regulates neutrophil activation, inhibits inflammatory macrophage infiltration, promotes the development of anti-inflammatory M2 macrophages, and enhances energy expenditure (60–63). Interestingly, insulin exposure is associated with reduced histamine release, enhanced LTC_4_ production, and increased resolvin D1 and E1 synthesis in RBL-2H3 cells (64). However, previous to this report, it was not known whether functional GPR40 and GPR120 were expressed by human MC. To our knowledge, this study is the first to show that LAD2 human MCs express *GPR40* and *GPR120* mRNA, and GPR120 protein, and that this expression is regulated by SCF. We further showed that PUFA inhibition of degranulation was diminished after blocking GPR120 with AH7614, suggesting GPR120 plays a role in n-3 PUFA's effect on MC activation. Additionally, we showed that TUG891 (a GPR120 agonist) and GW9508 (a GPR40/120 agonist) inhibited degranulation similarly to n-3 PUFA.

Although the mechanism of GPR120-inhibition of degranulation requires further study, it is possible that EPA binding of GPR120 activates G protein-coupled receptor kinase 6 and protein kinase C or increases the intracellular levels of cyclic adenosine monophosphate and subsequent activation of protein kinase A, all of which have been shown to inhibit MC activation (39–41, 65). However, the exact interplay between GPR120, FcεRI, and lipid rafts requires further investigation.

In summary, the current study indicates that n-3 PUFA reduces FcεRI localization into lipid rafts, inhibits FcεRI-mediated signaling pathway, and downregulates mediator release by LAD2 human MC, via an unknown mechanism associated with GPR120.

## Data Availability Statement

The original contributions presented in the study are included in the article/Supplementary Materials, further inquiries can be directed to the corresponding author/s.

## Author Contributions

XW designed, optimized, and performed the majority of the western blot, cell culture and mediator release experiments, and drafted the manuscript. RI performed the inhibitor experiments and contributed to the manuscript draft. BT designed the primers used in the qPCR experiments and performed the gene expression analysis experiments. SBA analyzed the inhibitor data, prepared some of the figures, and helped draft the manuscript. MK conceived of the project, obtained funding and resources, designed and optimized experiments, drafted the manuscript, and approved final submission of the manuscript for publication. All authors contributed to the article and approved the submitted version.

## Conflict of Interest

The authors declare that the research was conducted in the absence of any commercial or financial relationships that could be construed as a potential conflict of interest.
