# Supplementary figures and images for: Disrupted Lipid Raft Shuttling of FcεRI by n-3 Polyunsaturated Fatty Acid Is Associated With Ligation of G Protein-Coupled Receptor 120 (GPR120) in Human Mast Cell Line LAD2
Source: Front Nutr. 2020 Nov 26;7:597809. doi: 10.3389/fnut.2020.597809 (PMC7732685; doi:10.3389/fnut.2020.597809)

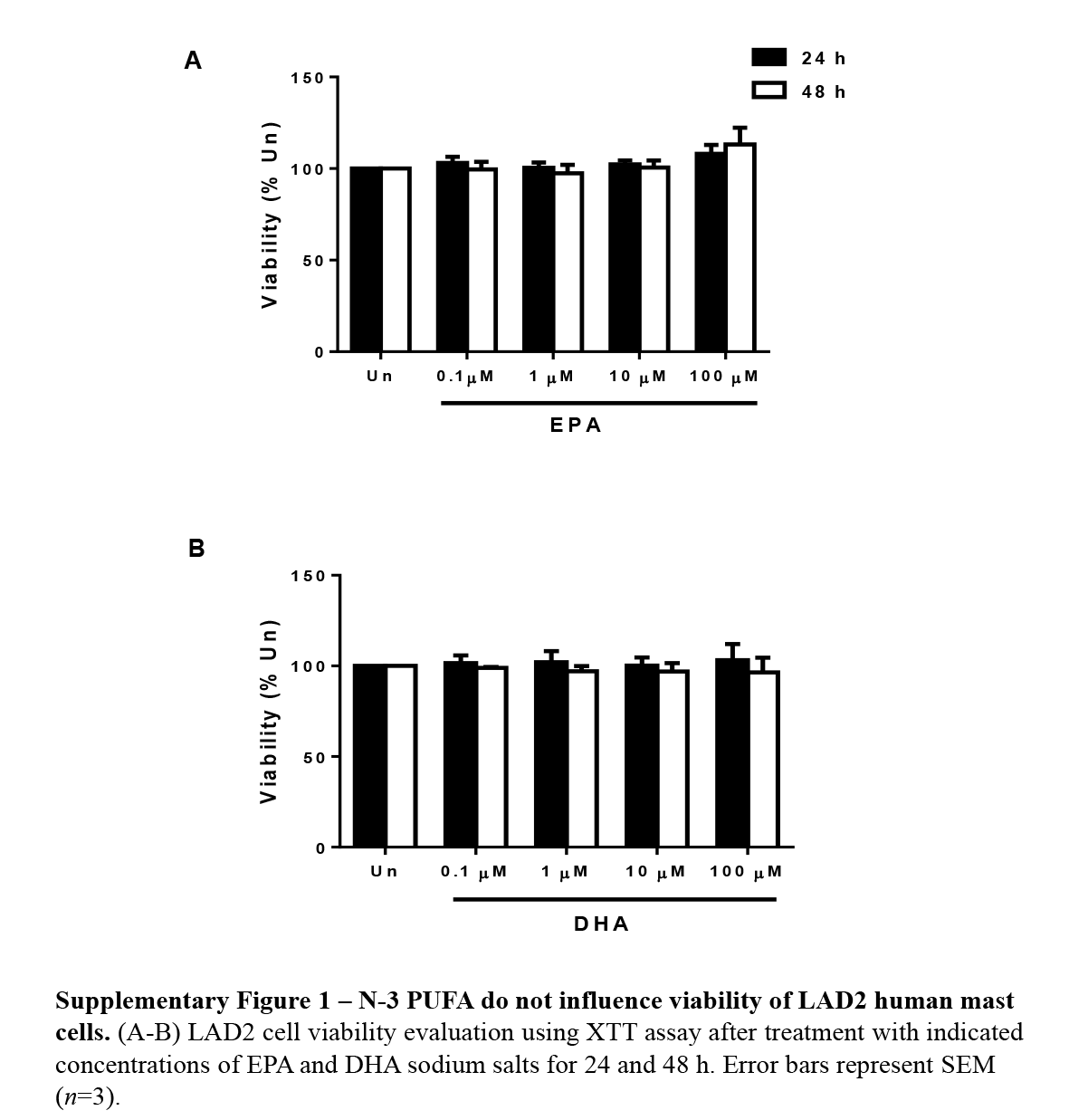

Supplement: Supplementary file 1 [file Image_1.TIF]
